# Pacemaker induced superior vena cava syndrome: a case report

**DOI:** 10.4076/1757-1626-2-6463

**Published:** 2009-07-29

**Authors:** Egambaram Senthilvel, Aphrodite Papadakis, Vikas Jain, Julia Bruner

**Affiliations:** 1 Department of Family Medicine, MetroHealth Medical Center, Case Western Reserve UniversityCleveland, OH 44109USA; 2 Department of Radiology, MetroHealth Medical Center, Case Western Reserve UniversityCleveland, OH 44109USA

## Abstract

**Introduction:**

Pacemaker induced superior vena cava syndrome is an unusual complication of pacemaker implantation. Endothelial damage caused by repeated trauma from the lead is thought to be responsible for the stenosis. Malignancy has been historically the most common etiology. However, the increase in use of indwelling venous catheters and cardiac pacemaker has resulted in more patients with superior vena cava syndrome of benign etiology.

**Case presentation:**

A 54-year-old female presented with recurrent spasm and swelling of the neck for the duration of two months. Pacemaker was implanted in 1997 for symptomatic third degree heart block. It was removed in 2007 due to recurrent infection at the lead site. Computed tomography of the chest and venogram were performed which showed stenosis at origin of the superior vena cava with some collateral circulation. She underwent angioplasty by the interventional radiology and is currently free of symptoms.

**Conclusions:**

Our case highlights a relatively uncommon complication of pacemaker. As a primary care physician, one should be aware of this unusual complication of pacemaker. Superior vena cava syndrome should be suspected in patients with history of pacemaker insertion who present to the primary care physician with neck spasm or neck swelling. Primary care physicians should also be aware balloon angioplasty is a reasonable primary intervention in selected patient population.

## Introduction

SVC syndrome was first described by William Hunter in 1757. Malignant diseases such as bronchial carcinoma, mediastinal lymphoma have been historically been the most common malignant etiology and mediastinal fibrosis has been most common cause of benign etiology.

However, the increase in use of indwelling venous catheters, cardiac pacemaker and implantable cardioverter defibrillator has resulted in more patients with SVC syndrome of benign etiology.

SVC thrombosis with or without stenosis from the pacemaker leads was described by Kosowsky and Barr in 1972 [1]. The incidence of this condition has been reported in the literature range from 1 in 650 patients to 1 in 3100 patients [2]. We report a case of SVC stenosis secondary to pacemaker insertion who underwent percutaneous transluminal angioplasty (PTA). As per our literature review, our case is a first report of its kind to indicate this complication occurred after removal of the pacemaker device.

## Case presentation

A 54 year old African American female presented with history of recurrent swelling of the neck and face for two months, mainly noticed in the morning time which resolved later in the day. She was also feeling tightening spasm in her neck for the same duration. She denied any shortness of breath, chest pain or cough. Clinical examination showed minimal edema of the face and prominent superficial veins of the chest wall and neck region. She had a history of pacemaker insertion in 1997 for complete heart block in an outside hospital (DDD pacemaker with triology DDR 2306L, pace setter pacemaker along with atriventricular leads). Because of persistent infection of the pacemaker site pacemaker/ wire was removed in 2007 by sternotomy in another outside hospital. Other past medical history is remarkable for hypertension, and obstructive sleep apnea.

**Figure 1.**
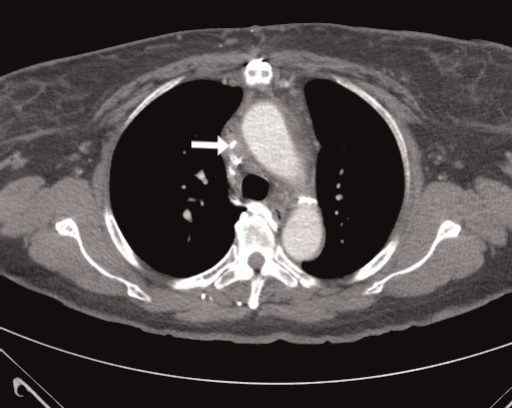
CT scan with contrast of the chest shows narrowing of the SVC.

**Figure 2.**
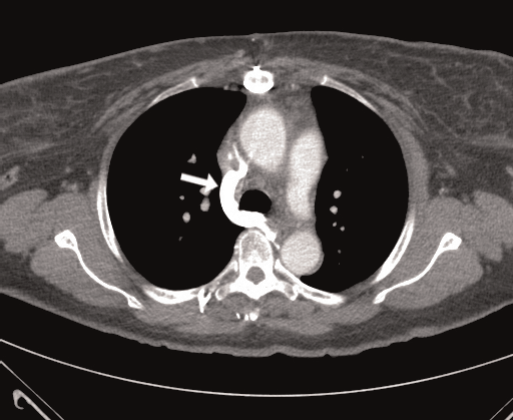
CT scan with contrast shows prominent caliber of the azygous vein due to collateral flow.

**Figure 3.**
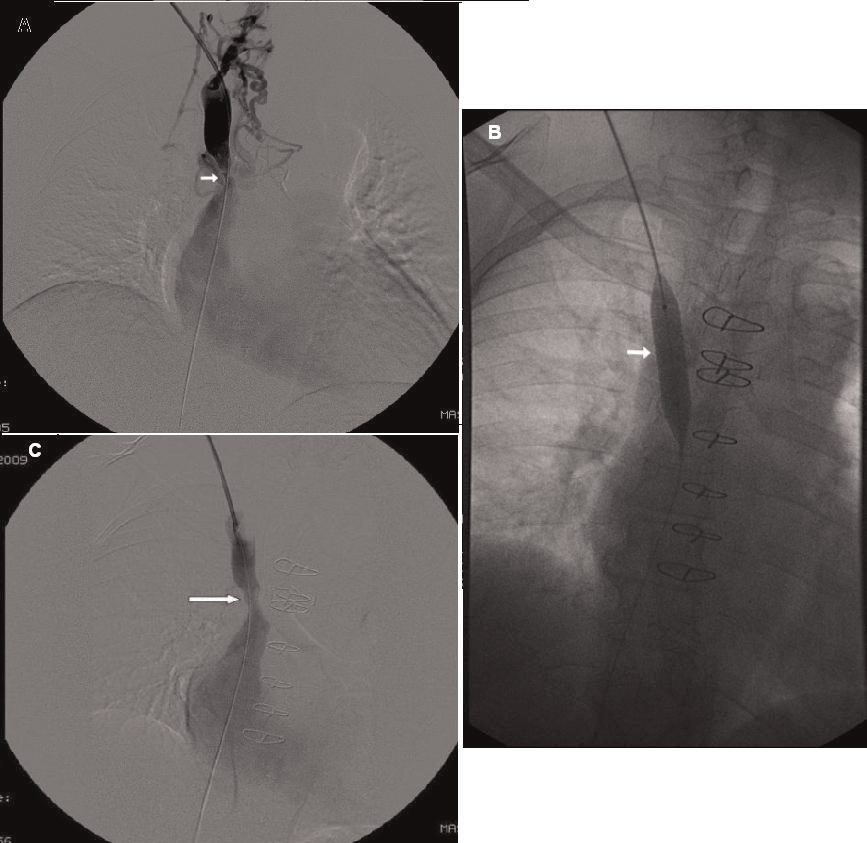
Venogram images of the SVC show, **(A)**. Stenosis with collateral circulation, **(B)**. Balloon angioplasty of the stenotic area, **(C)**. Improved flow of contrast in SVC after the angioplasty.

**Figure 4.**
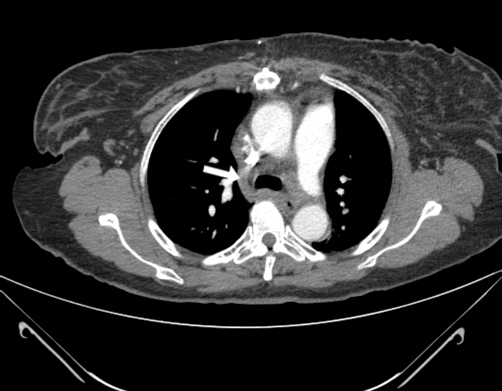
CT scan with contrast shows patent SVC at the site of previous stenosis after 2 months.

She has been taking metoprolol and lisinopril for her hypertension. She smokes 2 packs of cigarettes a day for the last 26 years and has significant history of alcohol intake. Current electrocardiogram showed normal sinus rhythm. Computed tomography (CT) of the chest was performed first (Figures 1, 2). An arm venogram confirmed SVC obstruction, with severe stenosis at the origin of SVC near the junction of azygous vein. Percutaneous endovascular procedures (PTA) was performed by right internal jugular vein approach by using 12 mm × 4 cm balloon which was advanced across the stenosis (Figure 3 A-C). Immediate post angioplasty venogram showed excellent flow identified in the SVC. The symptoms of superior vena cava syndrome began to improve immediately after the angioplasty and she continues to be free of symptoms 2 months after the procedure. A follow up CT scan of the chest was recently done during a hospitalization for community acquired pneumonia which showed patent stenotic area (Figure 4).

## Discussion

The pathogenesis of the SVC stenosis in pacemaker implanted patients is thought to be from endothelial disruption caused by repeated trauma from the leads and it usually occurs above the right atrium [3]. Deposition of fibrin on the surface of the leads can result in vessel wall inflammation, fibrosis, thrombus formation and eventually these changes lead to venous stenosis and occlusion [2]. Lead infection like our case, and retained leads increase the risk of thrombosis, stenosis and subsequent SVC syndrome. Lead material, caliber, and access site either by subclavian or cephalic did not have any impact on stenosis formation [4,5]. Stenosis due number of leads varies, but only one study report that increasing number of leads had been associated with more stenosis formation [6].

The most common clinical presentations are neck, facial and upper extremity swelling secondary to obstruction of blood flow in the SVC. Other commonly reported symptoms are dyspnea on exertion, orthopnea, headache, dizziness and visual changes. Head and neck swelling and chest wall venous collaterals are most commonly seen on clinical examination. However in most of the patients with pacemakers, swelling presents insidiously because of chronic process and the development of adequate collaterals. Because of high association of SVC syndrome with malignancy, a thorough history and physical examination should be performed in all cases. Venography is considered to be the gold standard for diagnosing venous obstruction [7], and is required before and after the vascular intervention. Spiral CT venography is also very helpful in diagnosing chest vein stenosis [8,9]. Magnetic resonance angiography (MRA) may also be considered when contrast venography is contraindicated.

Anticoagulation and thrombolytic agents are used for pacemaker induced venous thrombosis. It helps in maintaining the patency of venous collaterals and reduces thrombus propagation. Low molecular weight or unfractionated heparin is used along with warfarin, until the INR is in the therapeutic range (2 to 3). Warfarin is used for 3-6 months after the episode of thrombosis. Multimodal approach is evolving recently, which consists of heparinization, transcatheter thrombolytic therapy, followed by a minimum of three months of warfarin and balloon angioplasty for treatment of residual stricture [10]. Our patient had fibrous stenosis without underlying thrombus. Percutaneous endovascular procedures (PTA) have been mainly used for the management of SVC stenosis. Percutaneos balloon angioplasty was first reported by Sherry et al [11] in 1986. Use of percutaneous transluminal angioplasty alone [11-13] in SVC obstruction management has been described in previous studies.

Combination of PTA with different types of stenting [13,14,15] for the management of benign SVC stenosis has also been reported. These techniques are successful in eliminating or reducing the stenosis which relieves the symptoms of SVC obstruction and also helps pacemaker revision if needed. Our patient’s pacemaker was permanently removed prior to this presentation because of reversal of her complete heart block to normal sinus rhythm and her current EKG is showed normal sinus rhythm. Angioplasty does not adversely affect future open surgical reconstruction [16] and angioplasty has also been used for surgery failed patients. Open surgery the last resort of choice, mainly reserved for symptomatic occlusion that does not respond to percutaneous procedures.

## Conclusions

Superior vena cava syndrome is a recognized, uncommon complication of pacemaker implantation. Our case shows that this condition may occur even after the removal of the pacemaker. As a primary care physician, one should be aware of this unusual complication of the pacemaker. In patients with history of pacemaker insertion who presents to the primary care physician with neck spasm or neck swelling, one should suspect SVC syndrome. Primary care physician should also be aware of balloon angioplasty as a reasonable primary intervention in selected patient population.

## Abbreviations

CT: computed tomography
DDD: A pacemaker that senses and stimulates both atrium and ventricle. The stimulation rate is determined the maximum tracking rate and a minimum rate
DDD.R: A pacemaker that senses and stimulates both atrium and ventricle. The stimulation rate is governed by a maximum rate, the rate determined by a sensor and the minimum rate
EKG: electrocardiogram
INR: international normalized ratio
MRA: magnetic resonance angiography
PTA: percutaneous transluminal angioplasty
SVC: superior vena cava

## References

[bib-001] Kosowsky BD, Barr I (1972). Complications and malfunctions of electrical cardiac pacemaker. Cardiovasc Dis.

[bib-002] Rozmus G, Daubert JP, Huang DT, Rosero S, Hall B, Francis C (2005). Venous thrombosis and stenosis after implantation of pacemakers and defibrillators. J Interv Card Electrophysiol.

[bib-003] Bauset R (2002). Pacemaker-induced superior vena cava syndrome: a case report and review of management strategy. Can J Cardiol.

[bib-004] Da Costa SS, Scalabrini NA, Costa R, Caldas JG, Martinelli FM (2002). Incidence and risk factors of upper extremity deep vein lesion after permanent transvenous pacemaker implant: A 6-month follow up prospective study. Pacing Clin Electrophysiol.

[bib-005] Antonelli D, Turgeman Y, Kaveh Z, Artoul S, Rosenfield T (1989). Short-term thrombosis after transvenous permanent pacemaker insertion. Pacing Clin Electrophysiol.

[bib-006] Van Rooden CJ, Molhoek SG, Rosendaal FR, Schalji MJ, Meinder’s AE, Huisman MV (2004). Incidence and risk factors of early venous thrombosis associated with permanent pacemaker leads. J Cardiovasc Electrophysiol.

[bib-007] Bettman MA (1988). Noninvasive and venographic diagnosis of deep vein thrombosis. Cardiovasc Intervent Radiol.

[bib-008] Baldt MM, Zontsich T, Kainberger F, Fleischmann G, Mostbeck G (1997). Spiral CT evaluation of deep venous thrombosis. Semin Ultrasound CT MR.

[bib-009] Tello R, Scholz E, Finn JP, Costello P (1993). Sunclavian vein thrombosis detected with spiral CT and three-dimensional reconstruction. AJR Am J Roentgenol.

[bib-010] Kommareddy A, Zaroukian MH, Hassouna HI (2002). Upper extremity deep venous thrombosis. Sem Thromb Hemost.

[bib-011] Sherry CS, Diamond NG, Meyers TP, Martin RL (1986). Successful treatment of SVC syndrome by venous angioplasty. Am J Reontgenol.

[bib-012] Marzo KU, Schwartz R, Glanz S (1995). Early restenosis following percutaneous transluminal balloon angioplasty for the treatment of superior vena cava syndrome due to pacemaker- induced stenosis. Cathet Cardiovasc Diagn.

[bib-013] Kee ST, Kinoshita L, Razavi MK, Nyman URO, Semba CP, Dake MD (1998). Superior vena cava syndrome: treatement with catheter-directed thrombolysis and endovascular stent placement. Radiology.

[bib-014] Watkinson AF, Hansell DM (1993). Expandable Wallstent for the treatment of obstruction of the superior vena cava. Thorax.

[bib-015] Dodds GA, Harrison JK, O’Laoughlin MP, Wilson JS, Kissolo KB, Bashore TM (1994). Relief of superior vena cava syndrome due to fibrosing mediastinitis using the Palmaz stent. Chest.

[bib-016] Kalra M, Gloviczki P, Andrews JC, Cherry KJ, Bower TC, Panneton JM, Bjarnason H, Noel AA, Schleck C, Harmsen WS, Canton LG, Pairolero PC (2003). Open surgical and endovascular treatment of superior vena caval syndrome caused by nonmalignant disease. J Vasc Surg.

